# Pinworm Infestation Mimicking Crohns' Disease

**DOI:** 10.1155/2013/706197

**Published:** 2013-03-11

**Authors:** Joel Johansson, Simone Ignatova, Mattias Ekstedt

**Affiliations:** ^1^Medical School at the Faculty of Health Sciences, Linkoping University, 581 85 Linkoping, Sweden; ^2^Linköping University Hospital, Clinic for Pathology, Linkoping University Hospital, 581 85 Linkoping, Sweden; ^3^Gastroenterology and Hepatology, Department of Clinical and Experimental Medicine, Linköping University, Linkoping University Hospital, 581 85 Linkoping, Sweden

## Abstract

We here report a case of a young man who presented to his general practitioner with diarrhea. Inflammatory bowel disease was suspected and a colonoscopy showed aphthous lesions suggestive of Crohns' disease but biopsies revealed eggs of *Enterobius vermicularis.* When treated for this parasite, his symptoms were alleviated and a followup colonoscopy revealed a normal colon and distal ileum. Enterobius vermicularis is the most common parasite worldwide and has been attributed with many different presentations and pathologies. It is therefore necessary to maintain vigilance, even in high-income countries, in order to diagnose patients with one of the many atypical presentations of pinworms.

## 1. Introduction

In Scandinavia, the presence of aphthous ulcerations and erosions in the distal ileum and caecum is usually a manifestation of Crohns disease. However numerous differential diagnoses exist and histopathological confirmation is required in order not to give the patient the lifelong diagnosis of Crohns disease inaccurately.

*Enterobius vermicularis* is the most common helmintic parasite known, affecting all members of society regardless of age, gender, and social status [1]. They typically reside in the caecum, appendix and distal ileum, where they adhere to the mucosa [2]. Although many infections are asymptomatic, perianal itching, especially at night, is the most common symptom [1]. However there are a lot of atypical presentations described in the literature, for example, infections of the kidneys [3] and infections of the female genital tract [4] as well as many other presentations. 

Typically the diagnosis rests upon applying cellophane or scotch tape to the perianal skin in the morning, removing it, and detecting eggs using the microscope [1, 5]. The worms can however be seen during endoscopy [6], and both the worm and its eggs can be found in histological specimens [5, 7]. Once diagnosed the infection is eradicated with two doses of Mebendzole two weeks apart as well as hygienic measures [8].

We here present a case of a young man with an *Enterobius vermicularis* infection which mimics Crohns disease.

## 2. Case Presentation

A 22-year-old man presented to his GP with an exacerbation of diarrhea during the past two months. He reported that he usually had diarrhea once or twice a week mostly on weekends and on daytime normally beginning as abdominal cramps that was relieved by defecation. During the past two months, he had been having an increased amount of watery-thin diarrhea 5-6 days a week. He now had symptoms also during the night and with no relation to food intake. In addition abdominal cramps were sustained during the days of diarrhea. He had not been outside Sweden recently. Blood work included liver function tests, creatinine, ions, anti- transglutaminase and gliadin antibodies, thyroid function tests, a full blood count, stool culture and microscopy for cysts and worms. They all came back negative. He was diagnosed with IBS and his GP prescribed inolaxol and loperamide. 

He returned six months later with a history of one week of watery-thin diarrheas without mucus or blood. He also had abdominal cramps, nausea, and a fever of 38°C but no cardiopulmonary or mictuition problems. This time ESR was elevated as well as CRP and a blood count showed leucocytosis. He had not been traveling outside Sweden nor received antibiotics recently. Infectious enterocolitis was suspected and a stool culture was obtained.

Four days later the stool culture came back negative, his symptoms were slightly better, and CRP had dropped significantly. His GP suspected noninfectious bowel disease and ordered a quick test for fecal calprotectin. When it showed >60 *μ*g/g of feces he was referred for a colonoscopy. 

**Figure 1:**
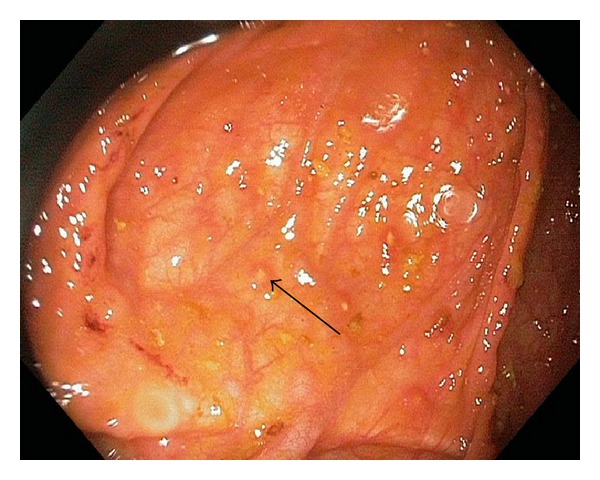
Multiple aphtous lesions (arrow) were found in the cecum and the ascending colon.

**Figure 2:**
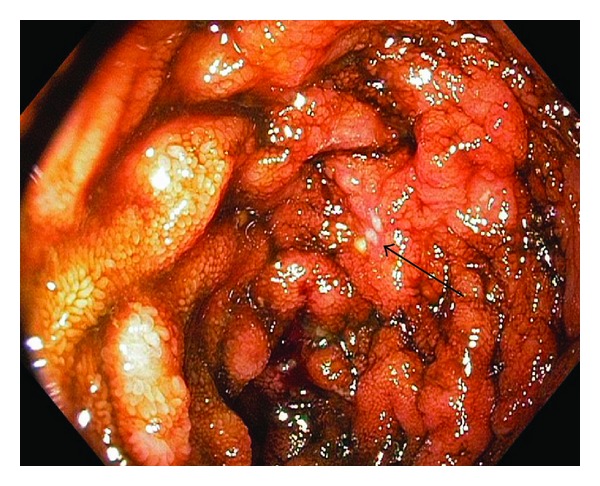
The mucosa of the distal ileum was swollen with friability. A cobblestone appearance with multiple aphtous lesions was seen (arrow).

The colonoscopy showed a normal colon up till mid transverse colon where erosions started to appear. These erosions increased distally up until the caecum (Figure 1), where aphthous ulcerations upon a erythematic base was seen. In the distal ileum cobblestone pattern was seen together with multiple erosions (Figure 2). The morphology suggested Crohns disease and multiple biopsies were taken and an MRI of the small bowel was scheduled; the patient was given Prednisolone which alleviated his symptoms.

**Figure 3:**
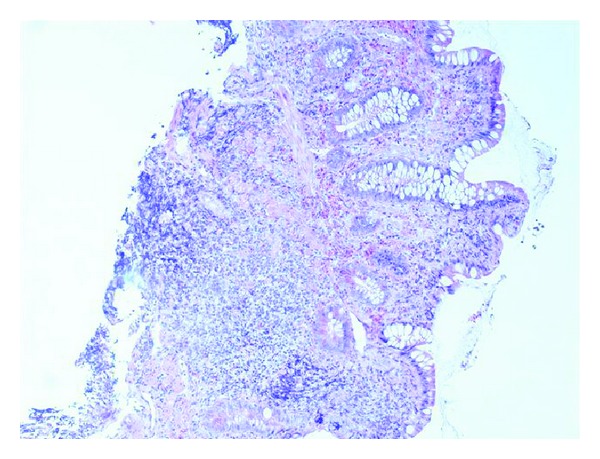
Irregular mucosa with inflammatory cell infiltrate composed of neutrophils and eosinophils which can be difficult to distinguish from preexisting inflammatory disorders.

**Figure 4:**
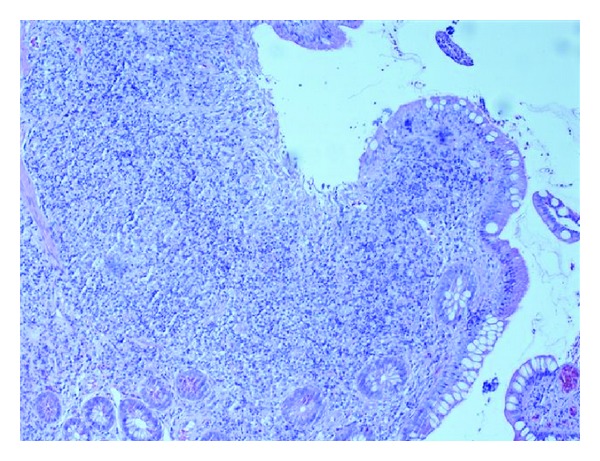
Broad mucosa with displaced glands and mucosal ulceration with mixed inflammatory nongranulomatous cell infiltrate in the lamina propria.

**Figure 5:**
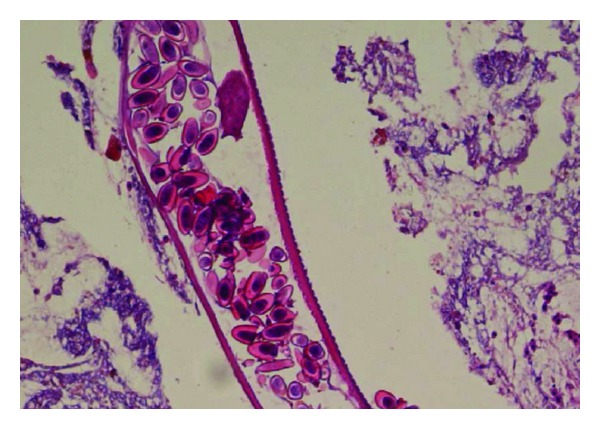
Longitudinal section through the larval stage of a female pinworm with numerous eggs.

When the biopsy results came back four weeks later they showed no crypt abscesses and no granulomas. In ileum lymphoid hyperplasia with germinal centers was found as well as focal neutrophilic infiltrates (Figures 3 and 4). In caecum and ascending colon a few spots with cryptitis were found. The most remarkable finding was a female larvae of *Enterobius vermicularis* with numerous eggs lying in the intestinal lumen (Figure 5). The patient was therefore given a single dose of Mebendazole with a second dose two weeks later. Three days after the first dose he still had an elevated fecal calprotectin and although he no longer had diarrhea he experienced nausea and vomiting. The MRI was normal and prednisolone was scaled out. 

A follow-up colonoscopy five months later revealed a macroscopically normal colon and distal ileum; biopsies were taken and they revealed lymphoid hyperplasia in the distal ileum and a normal caecum and colon. Fecal calprotectin was normalized during the following months and he remains symptom free.

## 3. Discussion

To our knowledge only three other cases of *Enterobius vermicularis* mimicking Crohns disease have been reported in the literature [2, 9, 10]. It is believed that *Enterobius vermicularis* cannot penetrate the intestinal mucosa unless there is some insult to the mucosal barrier. They are however known to be associated with colonic ulcerations but the question of causation remains unanswered. Using the Rome III criteria it is probable that our patient has IBS, but since IBS is not associated structural derangement of the colonic mucosa we have no indication of a previous insult to the mucosa. In two of the previous cases presented by Beattie et al. [9] and McDonald and Hourihane [2] a reasonable mechanism of mucosal damage was present. In the case presented by McDonald and Hourihane [2] a perforated appendix was deemed reason behind the symptomatic Enterobius vermicularis. Also in the case presented by Beattie et al. [9] *Campylobacter jejuni* was found in a stool culture and may have contributed to mucosal damage. The third case by Fernandez-Flores and Dajil [10] contains no explaining factor, as in the present case. The previous authors have described that the worms attach themselves to the mucosa using their heads [2]. This may cause the ulceration necessary for the pinworms to become invasive but the question remains unanswered. 

As in the case presented by Beattie et al. we could demonstrate a normal colon and distal ileum during a follow-up colonoscopy. In our case we could also show a normalized fecal calprotectin indicating that the mucosal inflammation had ceased. The prompt response of his symptoms to Prednisolone is probably due to the anti-inflammatory effect of the drug, limiting the mucosal inflammation, which gave him his symptoms. Despite prednisolone however his fecal calprotectin was elevated and did not start to drop until he received Mebendazole. This also highlights the importance of a correct diagnosis since Crohns disease is a chronic disease there is a possibility that future flare-ups could have been treated as Crohns disease. The diagnosis of Crohns disease must therefore rest upon stronger evidence than slight macroscopic changes and therapeutic evaluation since it could mask other causes such as an *Enterobius vermicularis* infection.

The question whether the worm we found in the lumen could have caused this inflammatory reaction does not have a definitive answer. In the case reported by McDonald and Hourihane worms were indeed present in the intestinal wall. However in the cases presented by Fernandez-Flores and Dajil the worm was found solely in the lumen covered by eosinophils. The worm was described as being found at the base of an ulcer in the case presented by Beattie et al. but the worm was deemed invasive by the authors. Lastly in a case where *Enterobius vermicularis* caused eosinophilic ileocolitis no worms were found in biopsy specimens obtained during colonoscopy instead the worm was found during stool examination [11].

In conclusion, the histopathological finding of a pinworm and the lasting remission after Mebendazole treatment lean towards the notion that *Enterobius vermicularis* infection and not Crohns disease is the cause of his symptoms. Since the patient responded well to Mebendazole and the inflammatory infiltrates found in the biopsy specimen were not typical for Crohns disease, we believe that our patients symptoms were caused by *Enterobius vermicularis*. It is today unknown if *Enterobius vermicularis* is an invasive pathogen or if structural damage is required for invasive infection. *Enterobius vermicularis* might be an important differential diagnosis to Crohns disease even in Scandinavia and other highly industrialized nations, since this easily treated parasite infection can greatly reduce the patients quality of life. It is necessary to maintain vigilance among pathologists since an *Enterobius vermicularis* infection can be very similar to Crohns disease during colonoscopy.
